# Psychological predictors, moderators, and mediators of treatment outcome among ART-treated women: a prospective study

**DOI:** 10.3389/frph.2025.1665920

**Published:** 2025-10-08

**Authors:** Maria Clelia Zurlo, Federica Vallone, Maria Francesca Cattaneo Della Volta

**Affiliations:** ^1^ Department of Humanities, University of Naples Federico II, Naples, Italy; ^2^ Dynamic Psychology Laboratory, University of Naples Federico II, Naples, Italy; ^3^ Department of Political Sciences, University of Naples Federico II, Naples, Italy

**Keywords:** assisted reproductive technology (ART), coping strategies, dyadic adjustment, infertility, psychological health, stress, treatment outcome

## Abstract

**Background:**

The value of combining infertility research in the psychological and medical fields, i.e., by exploring psychological factors associated not only with emotional adjustment to infertility experiences but also with treatment outcomes, is well-recognized. However, research that bridges these fields’ boundaries is still narrow, is featured by mixed evidence, and often lacks a valid theoretical framework to comprehensively explore and/or identify the multiple psychological risk and protective factors associated with assisted reproductive technology (ART) treatment outcomes. Thus, there is a need for further research in this promising field of reproductive health to foster a high-quality standard of care for infertile couples.

**Objective:**

Based on the infertility-related stress model, this prospective study recruited a group of infertile women at the beginning of their infertility treatment (T1) to explore and identify the psychological variables (predictors and moderating and mediating variables) associated with their treatment outcome (Failure/Success) at the 4-year follow-up (T2).

**Methods:**

At the beginning of their infertility treatments (T1, 2019), 120 women completed self-report measures that provided data on their background information (sociodemographic characteristics and type of diagnosis), infertility-related stressors, coping strategies, psychological health (State-Anxiety and depression), and the couple's dyadic adjustment. After 4 years (T2, 2023), medical records were collected to provide data on their treatment outcome (failure or success), duration of infertility, and number of treatment cycles. The main, moderating, and mediating hypotheses were tested using correlational analyses, logistic regression analyses, and Hayes’ PROCESS tool.

**Results:**

The couple's relationship concern stressor, the adoption of a positive attitude coping strategy, and a perceived couple's dyadic adjustment at T1 were significantly associated with treatment success at T2. Conversely, the social concern stressor, the adoption of an avoidant coping strategy, State-Anxiety, and depression at T1, along with a duration of infertility >3 years and a number of treatment cycles >4 at T2, were significantly associated with treatment failure. Duration of infertility, number of treatment cycles, the couple's dyadic adjustment, and the positive attitude and avoiding coping strategies emerged as significant moderating variables. Depression was a significant mediator in the association between the social concern stressor and the treatment outcome.

**Conclusions:**

This study’s findings provide evidence on the key psychological dimensions that should be assessed and addressed within multidisciplinary counseling interventions to support ART-treated women effectively throughout their infertility path and reduce the risk of treatment failure.

## 1 Introduction

In recent decades, infertility research in both the psychological and the medical fields has led to significant advancements in methodologies for the assessment of infertility/infertility experiences and the implementation of tailored evidence-based interventions (1–5).

In the psychological field, research has increasingly focused on the identification of specific factors that should be assessed/monitored throughout the infertility path to adequately support infertile couples in dealing with the emotional burden of such a diagnosis (6, 7). In this direction, recently, a valid multidimensional transactional model has been proposed, namely, the infertility-related stress model [I-STRESS model (8)]. This model allows researchers and practitioners to comprehensively assess a wide set of individual dimensions (sociodemographic characteristics and coping strategies), situational dimensions (fertility-related parameters and infertility-related stressors), and relational dimensions (the couple's dyadic adjustment) that have been empirically proven to play a significant role in predicting psychological health conditions in infertile patients (8–10). This model also accounts for the potential effects of the complex interplay among these multiple factors (i.e., by testing and identifying key predictors, moderators, and mediators), recognizing that—in real-life circumstances—infertile patients may be at higher or lower psychological risk depending on the simultaneous presence of risks and/or protective factors.

In the medical field, beyond the development of preventive strategies (i.e., detecting risk factors to reduce the chance of receiving an infertility diagnosis), research has increasingly targeted the identification of key clinical/laboratory biomarkers and physiological parameters that predict the success of assisted reproductive technology (ART) treatments (5, 11), thus providing the most accurate tools and advanced techniques to ensure that individuals/couples can achieve their parenthood desire.

However, although research in the medical and psychological fields has often traveled on parallel tracks, the value of integrating the two disciplines to provide a high-quality standard of care to infertile couples is well-recognized (12, 13). Therefore, a relatively narrow branch of research has begun to produce evidence crossing the fields’ respective boundaries, namely exploring multiple psychological factors that can promote or, conversely, hinder no longer the psychological adjustment to infertility experience alone but also the ART treatment success (14, 15). Specifically, research has highlighted the significant impact of specific sociodemographic characteristics [e.g., age, educational level, and employment status (16–20)] and fertility-related parameters [e.g., number of treatments (21)] on treatment success. Yet, above all, several studies have underlined the unfavorable impact of the increase of women' age (22–24) and of duration of infertility (25) on the likelihood to achieve a pregnancy [e.g., the following birth rates after 3 years of treatment have been found: 65% in patients aged 26–34 years; 60% in patients aged 35–37 years; 39% in patients aged 38–40 years; 15% in patients aged 41–42 years; 5% in patients aged 43–44 years; and no live births in patients aged ≥45 years and from the sixth cycle onwards (26, 27)]. Therefore, this evidence clearly encourages not only the timely recourse to ART treatments but also the quick repetition—whenever a treatment fails—of multiple *in vitro* fertilization (IVF) cycles to increase the chances of success [i.e., 75.8%–80.1% of couples achieved live birth after five cycles and 60% after 5 years of treatment (21)].

Nonetheless, the well-demonstrated psychological burden of repeated treatment failures (10, 28–31) makes it much more difficult for researchers and clinicians to “simply” recommend multiple interventions in rapid succession (with potential failures) to ensure success. In line with this, the branch of research that has attempted to identify factors predicting ART treatment success has not limited its focus to the investigation of background variables—on which an interdisciplinary support intervention can rarely be centered—but has also explored the association between the ART treatment outcome and psychological dimensions, such as perceived infertility-related stressors (14, 32–34), individual and relational dimensions [e.g., coping strategies (15, 22, 35–37), alexithymia (38, 39), and the couple’s dyadic adjustment and romantic attachment (36, 40, 41)], and psychological health conditions (42–48).

However, not only does the comparison of the findings from these studies still lead to the acknowledgement of mixed evidence that does not make it possible to clarify “whether” and “which” dimensions may represent predictors of ART treatments success, but these studies also explored the potential role of psychological dimensions independently, thus disregarding the potential of using a comprehensive transactional and multidimensional framework to simultaneously identify risk and protective factors (predictors, mediators, and moderators) linked to ART treatment outcome. Consequently, there is a clear need to develop further research in this relatively new field of study on reproductive health that examines the association between infertile patients’ psychological experiences and the outcome of medically assisted reproduction treatments.

In this direction, considering that the abovementioned research has emphasized the potential role of specific individual characteristics (sociodemographic characteristics and coping strategies), situational dimensions (fertility-related parameters and infertility-related stressors), and relational resources (the couple's dyadic adjustment) in influencing not only infertile patients’ psychological experience but also—albeit with mixed evidence—the ART treatment outcome, we hypothesized that the application of the I-STRESS model could be extended from the assessment of the predictors of patients’ psychological adjustment to infertility experience to achieve a greater understanding of the predictors of ART treatment outcomes.

Therefore, considering the research reported above, and based on the I-STRESS model (8), this prospective study recruited a group of infertile women at the beginning of their infertility treatment (T1) to explore and identify the psychological variables (predictors and moderating and mediating variables) associated with their treatment outcome (failure or success) at the 4-year follow-up (T2). Specifically, three main objectives, along with their respective hypotheses, were proposed and tested.

The first study objective (*Hypothesis One on Predictors; H1*) aimed to explore whether the I-STRESS model dimensions (i.e., sociodemographic characteristics, infertility-related parameters, adopted Coping Strategies, perceived couple's dyadic adjustment, and psychological health conditions assessed at T1 and duration of infertility and number of treatment cycles at T2) were significantly associated with the treatment outcome at T2 (failure or success). No prediction was made about the direction of the associations due to the mixed evidence reported in the literature.

The second study objective (*Hypothesis Two on Moderators; H2*) aimed to explore whether sociodemographic characteristics, infertility-related parameters, adopted coping strategies, and perceived couple's dyadic adjustment could serve as significant moderators in the association between the treatment outcome registered at T2 (failure or success) and the patient’s perceived infertility-related stressors and psychological health conditions, respectively.

The third and final study objective (*Hypothesis Three on Mediators; H3*) aimed to explore whether psychological health conditions could play a role as a significant mediator in the associations between the treatment outcome registered at T2 (failure or success) and the patient’s perceived infertility-related stressors, coping strategies, and the couple's dyadic adjustment dimensions, respectively.

**Figure 1:**
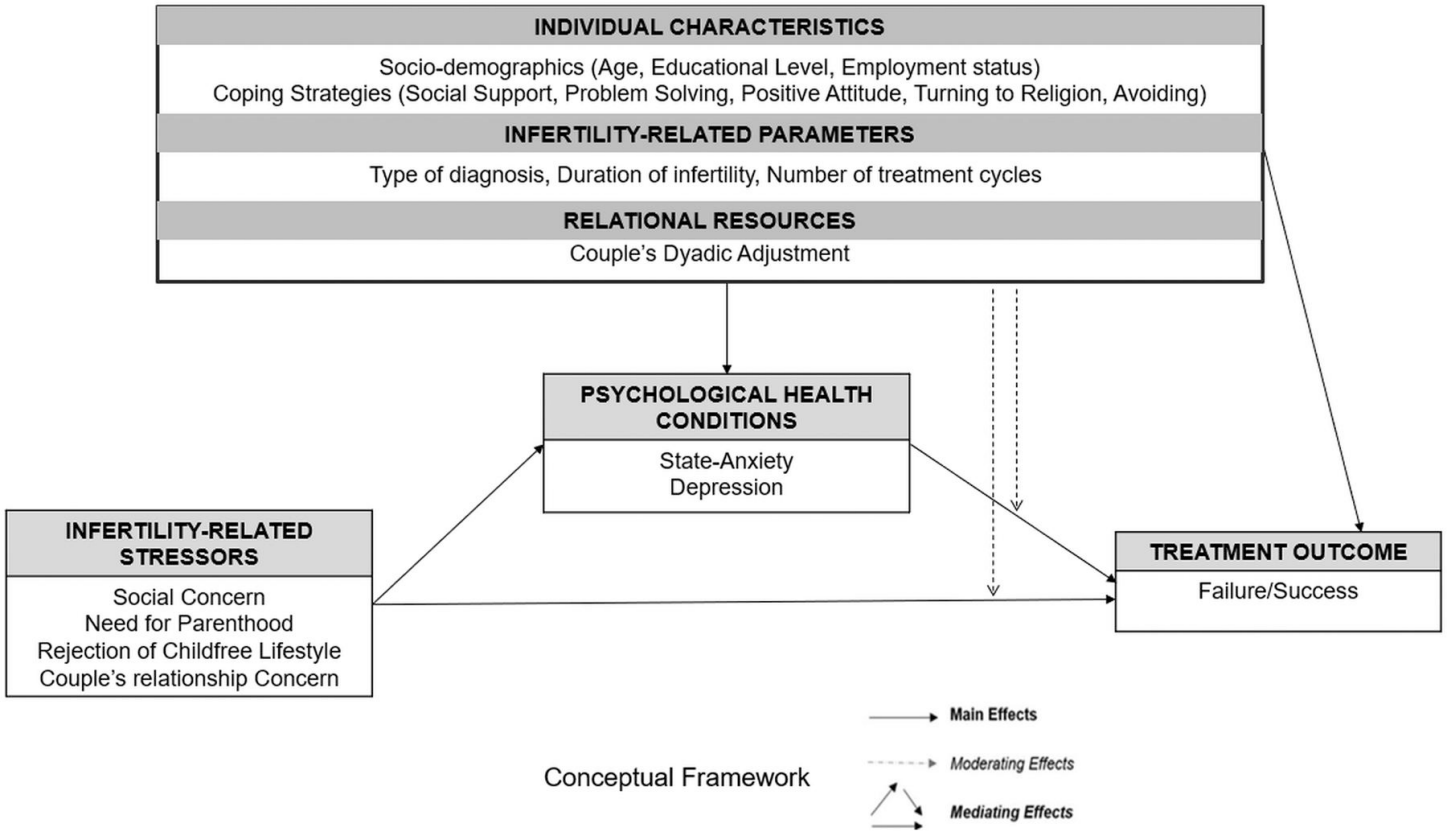
Conceptual framework.

The conceptual framework underpinning the study’s hypotheses is graphically illustrated in Figure 1.

## 2 Material and methods

### 2.1 Sampling and procedure

This prospective study enrolled 120 infertile women in 2019 (T1) at four centers of reproductive medicine in Italy. Preliminarily, chairpersons were asked to authorize the administration of a questionnaire in their centers, and after obtaining their authorization, the infertile women were directly invited to participate in the study. The eligibility criteria were as follows: (a) having received any diagnosis of infertility, i.e., due to female, male, or combined factors or unexplained; and (b) receiving their first ART treatment. Hence, 130 out of 373 women were found to be eligible and asked to complete a 25–30-min questionnaire (one session), with one of the authors always present to answer any queries raised by the participants. All the women were fully informed about the purpose of the study. They were assured about the confidentiality of the data, and they were informed that the data would be used only for the purpose of the research. The research was performed in accordance with the 1964 Helsinki Declaration and its later amendments or comparable ethical standards. The project was approved by the Ethical Committee of Psychological Research of the University of Naples Federico II (IRB: 34/2019). Every precaution was taken to protect the privacy of the participants and the confidentiality of their personal information.

**Figure 2:**
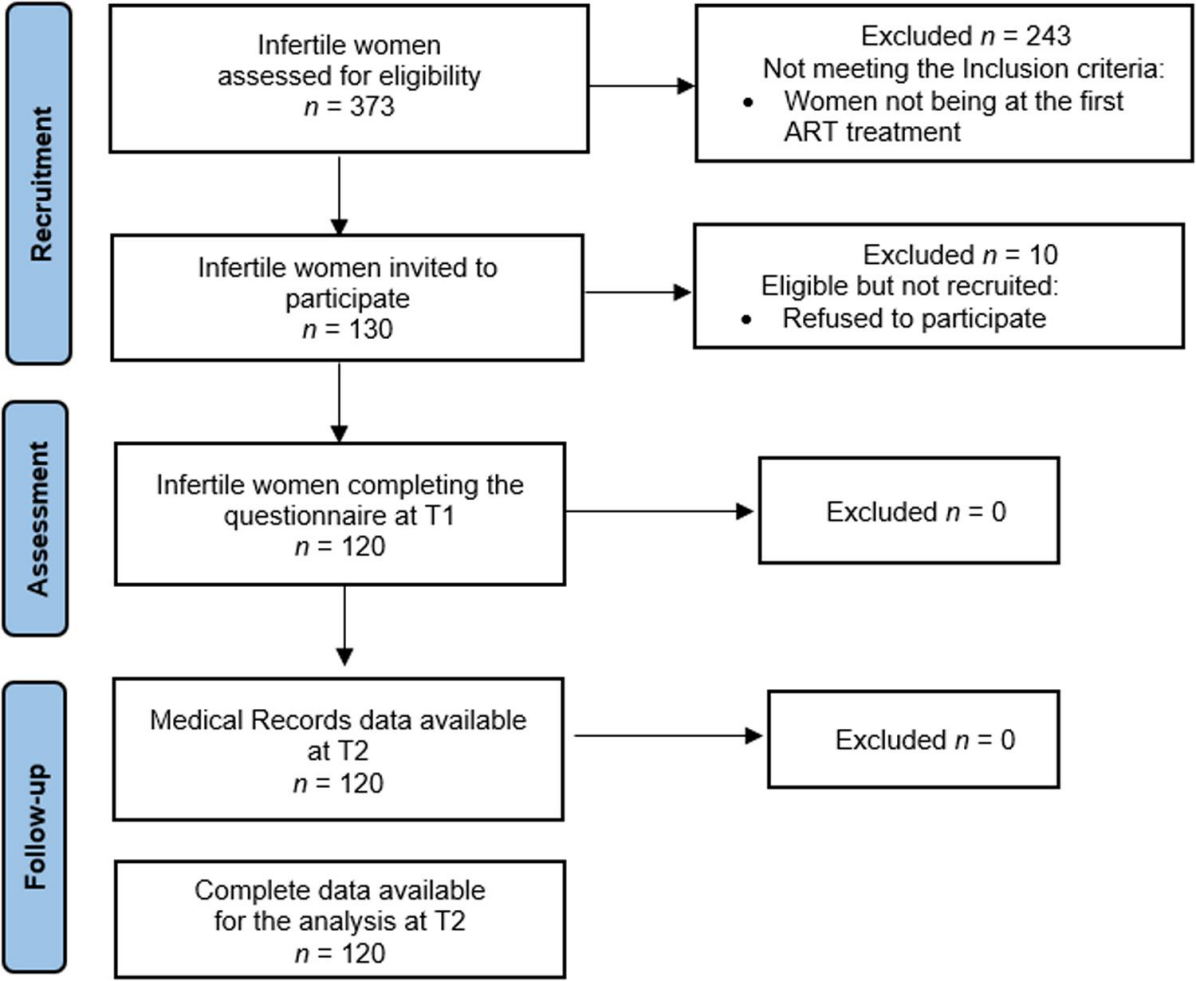
Flow diagram: participant recruitment and follow-up.

Overall, 120 women agreed to participate, provided written informed consent, and completed the questionnaire with no missing data (response rate of 92.31%). Figure 2 is the flowchart illustrating the participant recruitment process. All the women who participated were married in a heterosexual relationship and were diagnosed with primary infertility.

With respect to the study procedure, at T1, the women were asked to complete a questionnaire (I-STRESS questionnaire) to provide data on their background characteristics (sociodemographic characteristics and type of diagnosis), perceived infertility-related stressors, adopted coping strategies, perceived couple's dyadic adjustment, and psychological health conditions (State-Anxiety and depression). At T2 (i.e., 4 years after the assessment), for the purpose of the follow-up, chairpersons/physicians were directly contacted to match (using anonymized codes) the data collected at T1 with medical records containing the patients’ infertility treatment outcomes (failure or success), along with data on the duration of their infertility and the number of medical cycles they underwent. A live birth was considered the criterion for the success of the treatment.

### 2.2 Measures

At T1, the participants filled out the I-STRESS questionnaire (8), consisting of six sections, namely, (1) background information, (2) the Fertility Problem Inventory-Short Form (FPI-SF) (49) to assess *Infertility-related Stressors*, (3) the Coping Orientations to Problem Experienced-New Italian Version (COPE-NVI) (50) to assess the coping strategies adopted by the patients to deal with infertility, (4) the Dyadic Adjustment Scale (DAS) (51, 52) to assess perceived levels of the couple’s dyadic adjustment, (5) the State-Trait Anxiety Inventory (STAI-Y) (53, 54) to assess the patients’ perceived levels of State-Anxiety, and (6) the Edinburgh Depression Scale (EDS) (55, 56) to assess the patients’ perceived levels of depression.

At T2 (follow-up), medical records were used to collect data on the ART treatment outcomes (failure or success), duration of infertility (in years), and treatment cycles (number). A detailed description of the measures used, along with Cronbach's α and cut-off scores from the Italian validation studies of the measurement tools, is displayed in Table 1.

**Table 1:** I-STRESS model questionnaire: dimensions and description of measures.

Dimension	Measures	Variables
Sociodemographic and infertility-related characteristics	Single item questions (6 items)	Age (in years)
		Educational level (junior/middle school, senior school, college)
		Employment status (unemployed/employed)
		Type of diagnosis (male factor, female factor, combined factor, or unexplained)
		Duration of infertility (in years)
		Number of treatment cycles (number)
Infertility-related stressors	FPI-SF (49), 27 items, 6-point Likert scale	Social concern (10 items; Cronbach's *α* = 0.88; cut-off = 26.43)
		Need for parenthood (6 items; Cronbach's *α* = 0.88; cut-off = 26.54)
		Couples relationship concern (5 items; Cronbach's *α* = 0.70; cut-off = 11.62)
		Rejection of a childfree lifestyle (6 items; Cronbach's *α* = 0.77; cut-off = 26.73)
Coping strategies	COPE-NIV (50), 60 items, 5-point Likert scale	Social support (12 items; Cronbach's *α* = 0.88; cut-off = 27.7)
		Avoiding (16 items; Cronbach's *α* = 0.70; cut-off = 23.5)
		Positive attitude (12 items; Cronbach's *α* = 0.76; cut-off = 30.9)
		Problem solving (12 items; Cronbach's *α* = 0.83; cut-off = 32.0)
		Turning to religion (8 items; Cronbach's *α* = 0.85; cut-off = 22.7)
Dyadic adjustment	DAS (51, 52) 32 items	Dyadic consensus (13 items; Cronbach's *α* = 0.90)
		Affectional expression (4 items; Cronbach's *α* = 0.73)
		Dyadic cohesion (5 items; Cronbach's *α* = 0.86)
		Dyadic satisfaction (10 items; Cronbach's *α* = 0.94)
		Dyadic adjustment total (Cronbach's *α* = 0.93; cut-off = 115.7)
State-Anxiety	State Scale from the Italian version of the STAI-Y (53, 54) 20 items, 4-point Likert scale	State-Anxiety (Cronbach's *α* = 0.91; cut-off = 39.9)
Depression	EDS (55, 56) 10 items, 4-point Likert scale	Depression (Cronbach's *α* = 0.78; cut-off = 9)

### 2.3 Data analyses

The Statistical Package for the Social Sciences (SPSS; Version 21) was used for all the analyses. First, the women were prospectively grouped according to their treatment outcome at T2; thus, they were allocated to either the “treatment outcome success” group (i.e., the “Success” group) or to the “treatment outcome failure” group (i.e., the “Failure” group). Then, descriptive statistics were conducted, and potential statistically significant differences in the study variables were explored according to the treatment outcomes (success or failure). Differences were calculated using Student’s *t*-test for quantitative variables and the chi-square test for categorical/dichotomous variables. Given the multiple comparisons, the Bonferroni–Holm correction method was used to adjust the *p*-values (*α* = 0.05), thus reducing the risk of Type I error.

Second, to preliminarily explore the hypotheses related to the predictors (H1), moderators (H2), and mediators (H3), Spearman's correlations between the study variables were calculated. Therefore, to test H1 (i.e., I-STRESS model dimensions predicting the treatment outcome), a set of logistic regression analyses was run. Considering the interest in exploring the relationship between the I-STRESS model dimensions and the likelihood of treatment failure or success, and also taking into account the binary nature of the dependent variable explored in the present study (the treatment outcome was coded as Failure = 1, Success = 2), all the study variables were dichotomized by referring to the cut-off scores from the Italian validation studies reported in Table 1. Moreover, with respect to the fertility-related parameters, duration of infertility was dichotomized using the cut-off point (≤3 or >3 years) that has been repeatedly used in the literature (25, 31), while the number of treatment cycles was split at the median into low and high levels (≤4 and >4 cycles, respectively).

Afterward, to test H2 (i.e., the variables that moderate the relationship between the treatment outcome and infertility-related stressors and psychological health, respectively), a further set of logistic regression analyses was carried out. Finally, to test H3 (i.e., the psychological health conditions that mediate the relationship between the treatment outcome and the infertility-related stressors, coping strategies, and the couple’s dyadic adjustment dimensions, respectively), Hayes’ PROCESS tool for SPSS (Model 4) was used (57, 58). To verify the significance of the indirect effects, the Z Sobel test (59) and bias-corrected bootstrapped test with 5,000 replications to ensure a 95% confidence interval were conducted and reported (60).

## 3 Results

### 3.1 Preliminary data

Firstly, data obtained from the medical records collected at T2 and used to retrospectively group the 120 sampled women—revealed that 48.3% (n = 58) had a live birth (belonging to the “Success” group), 214 while 51.7% (n = 62) didn't have a live birth yet (belonging to the “Failure” group). Moreover, the data revealed statistically significant differences in the fertility-related parameters collected at T2 (Table 2). The duration of infertility in the Failure group was longer and more than one-half of the women in the Success group achieved a live birth after undergoing three to five treatment cycles. However, no live births were reported after five treatment cycles, with all the women who received five medical treatments belonging to the Failure group.

**Table 2:** The sociodemographic and infertility-related characteristics of the participants (*N* = 120) by treatment outcome.

Variable at T1	Treatment outcome at T2		*p-*value	Adjusted *p-*value^a^
	Treatment success *n* = 58 (48.3%)	Treatment failure *n* = 62 (51.7%)		
Age in years (mean ± SD)	34.6 ± 3.4	34.0 ± 3.4	0.925	1.000
Employment status, *n* (%)				
Unemployed	12 (52.2%)	11 (47.8%)	0.429	1.000
Employed	46 (47.4%)	51 (52.6%)		
Educational level, *n* (%)				
Junior middle school education	1 (7.7%)	12 (92.3%)	**0**.007**	0.077
Senior school education	33 (50.8%)	32 (49.2%)		
College education	24 (57.1%)	18 (42.9%)		
Type of diagnosis, *n* (%)				
Female factor	25 (53.2%)	22 (46.8%)	0.249	1.000
Male factor	20 (50.0%)	20 (50.0%)		
Combined factor	10 (55.5%)	8 (44.5%)		
Unexplained	3 (20.0%)	12 (80.0%)		
Variable at T2	Treatment outcome at T2		*p-*value	Adjusted *p-*value^a^
	Treatment success *n* = 58 (48.3%)	Treatment failure *n* = 62 (51.7%)		
Duration of infertility in years (mean ± SD)	3.0 ± 0.3	4.7 ± 0.8	**0**.000***	**0**.000***
Number of treatment cycles, *n* (%)				
<3	28 (68.3%)	13 (31.7%)	**0**.000***	**0**.000***
3–5	30 (81.1%)	7 (18.9%)		
>5	0 (0.0%)	42 (100.0%)		

Differences were calculated using Student’s *t-*test (mean ± standard deviations) and *χ*^2^-test [*N* (%)].

Statistically significant results are reported in bold.

^a^ Adjusted *p*-values were calculated using the Holm–Bonferroni correction method.

* *p* < 0.05; **p < 0.01; ***p < 0.001.

The mean differences in the study variables among the women in the Success and Failure groups are provided in Table 3. Regarding the infertility-related stressors, the data showed that the women in the Failure group had significantly higher levels of perceived stress related to the social concern dimension and significantly lower levels of stress related to the need for parenthood dimension than those in the Success group. Regarding the adopted coping strategies, the data showed that the women in the Failure group relied upon the problem-solving and avoiding coping strategies to a greater extent than those in the Success group. Finally, regarding psychological health conditions, the women in the Failure group reported statistically significantly higher levels of State-Anxiety than those in the Success group. There were no significant mean score differences by treatment outcome for perceived levels of stress related to rejection of childfree lifestyle and couple's relationship concern; for the use of the social support, turning to religion, and positive attitude coping strategies; for perceived couple's dyadic adjustment; and for depression.

**Table 3:** Means and standard deviations of the study variables at T1 according to treatment outcome at T2.

Variable at T1	Treatment outcome at T2		*p-*value	Adjusted *p*-value^a^
	Treatment success	Treatment failure		
	Mean ± SD	Mean ± SD		
Infertility-related stressors				
Social concern	24.97 ± 2.15	29.32 ± 3.52	**0**.000***	**0**.000***
Need for parenthood	26.59 ± 2.16	21.29 ± 3.49	**0**.000***	**0**.000***
Rejection of childfree lifestyle	22.14 ± 2.47	22.95 ± 2.86	0.220	1.000
Couple's relationship concern	12.86 ± 1.07	12.34 ± 1.67	**0**.044*	0.308
Coping strategies				
Social support	28.30 ± 2.55	25.72 ± 2.83	0.695	1.000
Problem solving	25.77 ± 3.21	26.39 ± 4.74	**0**.000***	**0**.000***
Positive attitude	28.88 ± 3.49	26.94 ± 2.88	**0**.010*	0.100
Turning to religion	20.72 ± 1.63	22.68 ± 1.89	**0**.031*	0.248
Avoiding	21.96 ± 1.80	26.31 ± 4.19	**0**.000***	**0**.000***
Couple's dyadic adjustment				
Dyadic adjustment	122.86 ± 7.22	119.54 ± 7.10	0.843	1.000
Psychological health conditions				
State-Anxiety	37.74 ± 1.73	38.99 ± 2.29	**0**.004**	**0**.048*
Depression	7.76 ± 1.01	9.09 ± 1.45	**0**.021*	0.189

Differences were calculated using Student’s *t*-test (mean ± standard deviations).

Statistically significant results are reported in bold.

^a^ Adjusted *p*-values were calculated using the Holm–Bonferroni correction method.

* *p* < 0.05; **p < 0.01; ***p < 0.001.

Considering the cut-off scores (Table 4), namely with respect to the number/percentages of women reporting low and high levels of perceived infertility-related stressors, coping strategies, the couple's dyadic adjustment, and psychological health conditions at T1 by treatment outcome at T2, the data revealed the following.

**Table 4:** Number and percentages of women reporting low and high levels of perceived infertility-related stressors, coping strategies, couple's dyadic adjustment, and psychological heath conditions at T1 by treatment outcome at T2.

Variable at T1	Treatment outcome at T2				*χ* ^2^
	Treatment success (*n* = 58)		Treatment failure (*n* = 62)		
	*N*	%	*n*	%	
Infertility-related stressors					
Social concern					
Low	43	74.1	15	24.2	
High	15	25.9	47	75.8	29.93***
Need for parenthood					
Low	54	93.1	61	98.4	
High	4	6.9	1	1.6	2.09
Rejection of a childfree lifestyle					
Low	56	96.6	57	91.9	
High	2	3.4	5	8.1	1.16
Couple's relationship concern					
Low	7	12.1	23	37.1	
High	51	87.9	39	62.9	**10.01** *
Coping strategies					
Social support					
Low	57	98.3	59	95.2	
High	1	1.7	3	4.8	0.90
Problem solving					
Low	53	91.4	51	82.3	
High	5	8.6	11	17.7	2.16
Positive attitude					
Low	36	62.1	52	83.9	
High	22	37.9	10	16.1	**6.98** *
Turning to religion					
Low	54	93.1	23	37.1	
High	4	6.9	39	62.9	40.88***
Avoiding					
Low	153	45.8	122	61.9	
High	121	44.2	75	38.1	2.58
Couple's dyadic adjustment					
Low	6	10.3	21	33.9	
High	52	89.7	41	66.1	**9.51** *
Psychological health conditions					
State-Anxiety					
Low	49	85.5	38	61.3	
High	9	15.5	24	38.7	**8.08** *
Depression					
Low	55	94.8	40	64.5	
High	3	5.2	22	35.5	**9.07** *

Differences were calculated using the *χ*^2^ test [*N* (%)].

Statistically significant results are reported in bold.

* *p* < 0.05. **p < 0.01. ***p < 0.001.

With respect to infertility-related stressors, the women in the two groups statistically differed in terms of stress related to social concerns and couple's relationship concerns, with the majority of the women in the Failure group reporting high levels of social concern (*n* = 47; 75.8%), while the majority of the women in the Success group reported high levels of couple's relationship concern (*n* = 51; 87.9%). Moreover, independent of the treatment outcome, more than 90% of the women reported low levels of need for parenthood and rejection of a childfree lifestyle. With respect to coping strategies, the data revealed a statistically significant difference in the use of the turning to religion coping strategy among the women in the Failure group (*n* = 39; 62.9%), while those in the Success group use positive attitude coping strategies to a greater extent (*n* = 22; 37.9%). With respect to the couple's dyadic adjustment, the data highlighted statistically significantly higher levels of perceived dyadic adjustment among the women in the Success group (*n* = 52; 89.7%). Finally, with respect to clinically relevant levels of psychological disease, the data highlighted a statistically significantly higher number of women reporting clinical levels of State-Anxiety (*n* = 24; 38.7%) and depression (*n* = 22; 35.5%) among those in the Failure group.

Before hypothesis testing, intercorrelations among study variables were calculated and are presented in Table 5. The data supported the testing of the main predictor (H1), moderating (H2), and mediating (H3) hypotheses.

**Table 5:** Intercorrelations among the study variables (*N* = 120).

Study variables	1	2	3	4	5	6	7	8	9	10	11	12	13	14	15	16	17	18	19	20	21	22
Sociodemographic characteristics																						
1. Age	1																					
2. Employment status	0.20*	1																				
3. Educational level	0.16	–0.02	1																			
Infertility-related parameters																						
4. Female diagnosis	−0.11	−0.04	0.07	1																		
5. Male diagnosis	0.02	−0.01	0.13	−0.57**	1																	
6. Combined diagnosis	−0.08	0.07	−0.03	−0.33**	−0.29**	1																
7. Unexplained diagnosis	0.20*	0.00	−0.2*	−0.31**	−0.28**	−0.16	1															
8. Duration of infertility	0.01	0.04	−0.30**	−0.09	0.00	−0.03	0.16	1														
9. Number of treatment cycles	−0.11	−0.02	−0.38*	−0.04	−0.02	0.03	0.05	0.62**	1													
Infertility-related stressors																						
10. Social concern	−0.11	0.08	−0.39*	−0.20*	0.20*	−0.09	0.10	0.56**	0.50**	1												
11. Need for parenthood	0.08	−0.01	0.43**	0.07	−0.00	0.12	−0.23*	−0.66**	−0.67**	−0.55**	1											
12. Rejection of a childfree lifestyle	−0.02	−0.03	0.06	−0.02	0.10	−0.03	−0.07	0.02	−0.13	0.08	0.12	1										
13. Couple’s relationship concern	0.09	0.02	0.29**	−0.01	0.06	−0.00	−0.07	−0.27**	−0.43**	−0.20*	0.42**	0.02	1									
Coping strategies																						
14. Social support	0.01	0.13	0.31**	−0.07	0.05	0.10	−0.08	−0.50**	−0.52**	−0.33**	0.54**	0.05	0.31**	1								
15. Problem solving	0.17	0.04	0.29**	−0.03	0.03	0.10	−0.09	−0.25**	−0.45**	−0.05	0.29**	0.12	0.32**	0.43**	1							
16. Positive attitude	0.20*	0.02	0.19*	−0.03	0.07	−0.01	−0.09	−0.25**	−0.29**	−0.25**	0.30**	0.08	0.16	0.08	0.05	1	*					
17. Turning to religion	−0.31**	−0.03	−0.36**	−0.09	−0.06	−0.00	0.23*	0.54**	0.57**	0.40**	−0.60*	−0.02	−0.29**	−0.46**	−0.53**	−0.26**	1					
18. Avoiding	−0.19*	0.10	−0.53**	−0.04	0.09	−0.20*	0.15	0.53**	0.71**	0.53**	−0.65**	−0.10	−0.31**	−0.56**	−0.39**	−0.24**	0.55**	1				
Relational resources																						
19. Couple’s dyadic adjustment	0.14	0.03	0.30**	−0.06	0.04	0.03	−0.00	−0.14	−0.17	−0.38**	0.17	−0.01	0.16	0.00	0.04	0.05	−0.21*	−0.19*	1			
Psychological health conditions																						
20. State-Anxiety	−0.03	0.16	−0.09	−0.09	0.07	0.00	0.01	0.28**	0.17	0.18*	−0.24**	−0.02	−0.09	−0.20*	−0.09	−0.03	0.13	0.32**	0.08	1		
21. Depression	−0.09	−0.03	−0.38**	−0.10	−0.03	0.01	0.19*	0.49**	0.38**	0.34**	−0.44**	−0.00	−0.19*	−0.44**	−0.12	−0.24**	0.41**	0.38**	−0.23*	0.13	1	
Treatment outcome																						
22. Treatment success	0.07	−0.03	0.25**	0.07	0.02	0.03	−0.18*	−0.85**	−0.47**	−0.61**	0.67**	−0.11	0.18*	0.43**	0.04	0.26**	−0.50**	−0.46**	0.23*	−0.26**	−0.47**	1

Spearman's correlation values. **p < 0.01; *p < 0.05.

### 3.2 Hypothesis 1 (H1)—predictors

The findings from the logistic regression analyses highlighted that the women reporting high duration of infertility, high number of treatment cycles, high levels of social concern, recurring to a greater extent to avoiding coping strategy, and reporting clinical levels of State-Anxiety and depression were statistically significantly less likely to be in the Success group. Conversely, the women who reported a high level of couple's relationship concern, greater use of the positive attitude coping strategy, and higher perceived levels of couple's dyadic adjustment were statistically significantly more likely to be in the Success group.

### 3.3 Hypothesis 2 (H2)—moderators

With respect to interaction analyses (Table 6), the data revealed that specific fertility-related parameters (i.e., duration of infertility and number of treatment cycles) and coping strategies (i.e., positive attitude and avoiding) and perceived Couple's Dyadic Adjustment were moderators in the association between the treatment outcome and the infertility-related stressors and Psychological Health conditions, respectively. Specifically, a longer duration of infertility and a higher number of treatment cycles significantly decreased the likelihood of belonging to the Success group among women with high levels of perceived social concern and clinical depression, i.e., those who were already at high risk.

**Table 6:** Predictors and moderators of treatment outcome (success) *N* = 120.

Treatment outcome at T2 (Success)		
Predictors	OR	95% CI
Duration of infertility at T2	0.02	0.00–0.16***
Number of treatment cycles at T2	0.16	0.07–0.38***
Social concern at T1	0.11	0.05–0.25***
Couple's relationship concern at T1	4.30	1.67–11.03**
Avoiding coping strategy at T1	0.14	0.06–0.31***
Positive attitude coping strategy at T1	3.18	1.35–7.51**
Dyadic adjustment at T1	4.44	1.64–12.01**
State-Anxiety at T1	0.29	0.12–0.70**
Depression at T1	0.10	0.03–0.35***
Moderators		
Social concern at T1 × duration of infertility at T2	0.04	0.01–0.10***
Social concern at T1 × number of treatment cycles at T2	0.03	0.01–0.11***
Social concern at T1 × avoiding at T1	0.04	0.02–0.15***
Social concern at T1 × positive attitude at T1	0.30	0.16–0.56***
Social concern at T1 × dyadic adjustment at T1	0.34	0.15–0.76*
Couple's relationship concern at T1 × positive attitude at T1	3.10	1.27–7.55**
Couple's relationship concern at T1 × avoiding at T1	0.45	0.29–0.72***
Couple's relationship concern at T1 × dyadic adjustment at T1	4.49	2.02–9.94***
Depression at T1 × duration of infertility at T2	0.03	0.00–0.25***
Depression at T1 × number of treatment cycles at T2	0.04	0.00–0.31***
Depression at T1 × positive attitude at T1	2.28	1.03–4.94***

Only significant associations were displayed.

* *p* < 0.05; **p < 0.01; ***p < 0.001.

Similarly, the greater the use of the avoiding coping strategy, the lower the likelihood of belonging to the Success group; however, this was found not only among the women with a high perceived level of social concern, but also among the women with a high perceived level of couple's relationship concern. The latter had a statistically significantly higher chance of belonging to the Success group when not extensively relying on escape, detaching, or avoiding strategies. In contrast, the greater the use of the positive attitude coping strategy, the higher the likelihood of belonging to the Success group, not only among women with a high perceived level of couple's relationship concern but also among the women who reported clinical levels of depression. The latter had a statistically significantly lower chance of belonging to the Success group when not extensively relying on positive reframing.

Furthermore, having a high perceived level of couple’s dyadic adjustment was associated with a higher likelihood of belonging to the Success group among the women with a high perceived level of couple's relationship concern. Nonetheless, neither the positive attitude coping strategy nor the couple's dyadic adjustment, which represented significant resources, was able to impact the association between the treatment outcome and social concern, resulting in a statistically significant lower chance of success. Finally, despite evidence of the detrimental effect of State-Anxiety on treatment success (main predictor), no interaction effects with either coping strategies or dyadic adjustment dimensions were found.

### 3.4 Hypothesis 3 (H3)—mediators

**Figure 3:**
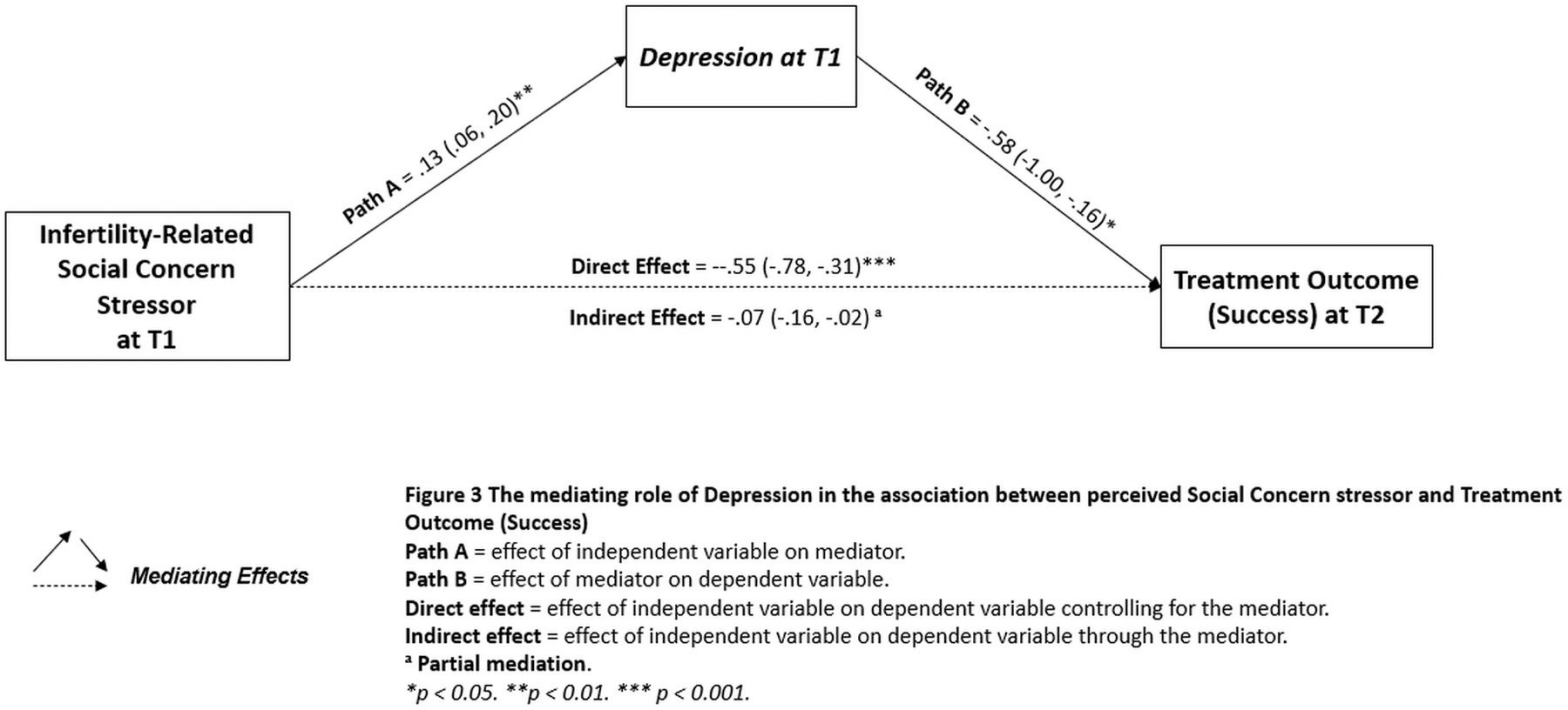
The mediating role of depression in the association between the perceived social concern stressor and treatment outcome (success).

Hayes’ PROCESS tool for SPSS was used to investigate whether psychological health conditions mediate the associations between the treatment outcome and infertility-related stress dimensions, coping strategies, and couple's dyadic adjustment dimensions, respectively. The data revealed that depression partially mediated the association between social concern and treatment outcome (Nagelkerke *R*^2^ = 0.12, *p* < 0.001) (Figure 3). Specifically, the data revealed a statistically significant direct effect of social concern on treatment outcome (Effect = −0.55; SE = 0.12; Z = −4.59; CI = −0.78 to −0.31; *p* < 0.001), with the indirect effect of *social concern* through *depression* (Effect = −0.07) being statistically significant since the confidence interval did not contain zero (CI = −0.16 to −0.02), and the Sobel test was significant (Z = 2.63, *p* = 0.008).

## 4 Discussion

By applying the I-STRESS model (8), this study enrolled a group of infertile women at the beginning of their infertility treatment (T1) to explore and identify the psychological variables (predictors, moderators, and mediators) associated with treatment outcome (failure or success) at the 4-year follow-up (T2).

Firstly, medical records of sampled women revealed that, 4 years after the first infertility treatment, 48.3% of the women achieved a live birth, while 51.7% of the women did not. Furthermore, most of the women who had undergone three to five treatment cycles achieved motherhood (81.1%), while all the women who had undergone more than five cycles in the 4 years were still childless (100%). This is somewhat in line with medical research suggesting that 75.8%–80.1% of couples achieve live birth after five cycles (21), with no live births from the sixth cycle onwards—even if this latter finding concerned older patients (≥45 years old) (26, 27).

However, overall, these findings seem to suggest an important aspect that should be carefully accounted for within multidisciplinary interventions implemented in medical settings, namely, these data corroborated the use of timely ART treatments (by mainly considering the factor of ageing); yet, at the same time, they also discourage the excessively rapid repetitions of ART treatment cycles (i.e., about two cycles—thus two failures *per* year). This could, indeed, hinder the possibility for women to have adequate time to recover both physically and mentally after a treatment failure, potentially exacerbating the suffering and feelings of hopelessness linked to multiple, rapidly repeated losses and grief.

In line with this, preliminary findings about psychological health conditions further emphasized the noteworthy psychological risk to which women still facing infertility treatments (and multiple failures) are exposed to. Indeed, the data underlined that the women in the Failure group reported—at the beginning of the treatment path (T1)—higher percentages of clinical levels of State-Anxiety (38.7%) and depression (35.5%) when compared to those who then achieved motherhood (State-Anxiety: 15.5%; depression: 5.2%). Timely multidisciplinary interventions are therefore required that put the overall reproductive health needs of infertile patients at their center, namely, both their psychological health and their desire to achieve parenthood.

Thus, considering the core hypotheses of our study and, specifically, with respect to H1 (predictors of treatment outcome), this study’s findings revealed that a wide set of specific risk factors (i.e., long duration of infertility, high number of treatment cycles, high perceived level of the social concern stressor, greater use of the avoiding coping strategy, and clinically relevant levels of State-Anxiety and depression) and protective factors (i.e., high perceived level of the couple's relationship concern stressor and couple's dyadic adjustment and greater use of the positive attitude coping strategy) were associated with the patients’ treatment outcomes, thus contributing to the international debate featuring the mixed research on the topic, but also indicating the potential of all the I-STRESS model dimensions—including psychological health conditions—to extend our knowledge of the factors associated with infertility treatment outcomes. Specifically, considering the risk factors, in line with our descriptive data on fertility-related parameters, this study’s findings corroborated previous studies warning that the psychological burden related to duration of infertility and repeated failures may affect not only psychological health conditions (31, 61), but also the treatment outcome (25, 62).

Moreover, the data provided further evidence supporting previous research highlighting that women’s use of avoiding coping strategies (36) and the presence of psychopathological conditions related to anxious and depressive symptoms (45, 48) may represent significant risk factors associated with treatment failure, thus fully corroborating the large body of converging evidence on the inherent link between psychological suffering and treatment outcome (14), yet also suggesting the need to offer multidisciplinary interventions in clinical settings to promote reproductive health effectively. This should be done by providing infertile couples with adequate resources and tools for dealing with the physical and psychological burdens experienced throughout the course of treatment.

Nonetheless, considering the risk factors, the data originally highlighted that perceived stress related to social concern was the sole infertility-related stressor significantly associated with the likelihood of treatment failure. This finding, in line with the large amount of evidence on the association between stress and IVF failure (63), is, however somewhat in contrast with research that has reported evidence on the main role of the personal pressures/needs and marital/sexual sources of stress on treatment outcome (14, 33), suggesting the need to carefully take into account the role of social pressures, which could significantly hinder the chance of treatment success.

From this perspective, this evidence on the specific detrimental effect of social concern could be discussed by taking into account infertility research exploring parenthood motives. In particular, it has been suggested that social pressure (i.e., motivation to meet the demands of society/family/relative/friends) and, accordingly, patients’ willingness to correspond to the parenting expectations of society, not only could expose them to higher psychological risk when a treatment fails (64), but could even play a role in compromising IVF treatment success (36).

Furthermore, this finding can also be discussed by considering the inherent nature of the infertility-related social concern stressor*.* This features the following characteristics, namely, high sensitivity to comments, intense feelings of social isolation, and alienation from family or peers. From this perspective, it could be hypothesized that the perceived shame, the fear of disclosing one’s condition, and the social isolation/alienation could result in women relying to a lesser extent on social support, with the latter linked to better psychological adjustment to IVF (65) and to a higher likelihood of treatment success in infertility research (36). However, notwithstanding the clear potential of social factors influencing not only the psychological aspects of infertility experience but also the treatment outcome, further research is needed to shed light on the role of the social domain within multidisciplinary infertility research.

Regarding H1 and the protective factor findings, the data supported the well-demonstrated positive role of specific individual and relational resources, such as the use of positive attitude coping strategies (37) and perceived marital satisfaction/couple’s dyadic adjustment (40, 66), not only in terms of fostering the psychological adjustment to infertility experience but also in relation to the treatment success. Furthermore, interestingly, the data revealed the positive role of the infertility-related couple's relationship concern stressor. From this perspective, whereas previous research has highlighted the detrimental effect of marital/sexual stressors on treatment outcome (14, 33)—proposing the explicative hypothesis that patients who experienced marital conflict were less likely to undergo treatments and, thus, less likely to achieve a pregnancy—the findings in this study suggested a different, and potentially favorable, role for couple's’ relationship concern.

Nonetheless, to shed light on findings concerning predictors (H1), and in line with the adoption of the transactional multidimensional perspective underpinning the I-STRESS model (8), there was a need to go further into our core research hypotheses by exploring psychological moderating (H2) and mediating variables (H3). This, indeed, provided further insight and a more complex understanding of how the interplay among psychological dimensions was associated with the treatment outcome. Thus, with respect to the moderating effects (H2), and specifically considering the moderators of the couple's relationship concern stressor, the results highlighted that the women who perceived higher stress related to this latter stressor and, at the same time, tried to distance themselves from stressful situations (using avoiding coping strategies), were at higher risk for treatment failure. In contrast, the co-presence of individual resources, such as the use of strategies centered on a positive re-appraisal of the stressful situation (i.e., a positive attitude coping strategy), and/or relational resources, such as the perceived couple's cohesion, consensus, gratification, and satisfactory intimacy (i.e., the couple's dyadic adjustment), were linked to an increased chance of achieving parenthood.

Therefore, it is sound to hypothesize that the role of the couple's relationship concern stressor as a risk or protective factor may depend on the simultaneous presence of further key dimensions. In other words, when women perceived high stress related to the detrimental effects on their relationship due to the burden of dealing with the infertility journey, but they also perceived themselves to belong to a solid and cohesive dyad, this could represent a “wholesome concern” that plays a protective role, fostering psychological health and better adjustment to the treatments. In the same direction, regarding the patient’s concerns about the couple's life, when they are openly expressed, faced, and reappraised (i.e., low use of the avoiding coping strategy and high use of the positive attitude coping strategy), they could also play a role as a motivational drive for dealing with infertility treatment without the desire for parenthood prevailing over the couple's relationship, thus—once again—fostering a better adjustment to treatments and potentially promoting its success.

In contrast, when considering the infertility-related social concern stressor, the co-presence of individual and relational resources was not able to counteract its detrimental effects. Rather, the data suggested that the co-occurrence of a long duration of infertility and an increased number of treatment cycles and the adoption of passive coping strategies (i.e., avoiding) should be evaluated carefully. Indeed, the cumulative presence of these multiple risk factors was found to be associated with an even lower chance of treatment success. From this perspective, these data further underlined the key and unique detrimental role of the social concern stressor. In this regard, the literature suggests that when a patient’s motivation to achieve parenthood is extrinsic—mainly driven by environmental pressure or by the need to meet expectations from society and/or family—their ability to cope with stress linked to infertility is lessened (67), and this may have a significant effect on their reproductive functions (36).

Nonetheless, the findings from moderation analyses (H2) revealed another key aspect to be accounted for when defining interventions. Specifically, considering depression, on the one hand, the significant increase in risk in cases of prolonged infertility and repeated cycles of attempts and failures (i.e., recurrent losses, grieving, feelings of defeat, and loss of hope) was confirmed once again; however, on the one other hand, the women who reported clinically relevant levels of depression but also relied on a positive attitude coping strategy were more likely to belong to the Success group. These findings should be carefully considered since they suggest the hypothesis that even in the cases of more intense depressive states, fostering a reframing strategy among these women (re-elaboration and re-appraisal within psychological counseling interventions) could increase the chance of success, thus creating a virtuous circle that could potentially reduce—by breaking the vicious circle of treatment failures—the depressive symptoms.

In this direction, by combining these latter findings with those from the mediation analyses (H3), it is possible to achieve a greater understanding of this complex portrait, providing further useful clinical insights for the development of supportive counseling interventions. Specifically, this study’s findings revealed that depression partially mediated the association between one of the key risk factors that emerged in this study, namely social concern, and treatment outcome. This finding could provide a better understanding of the underlying pathways of the relationship between social factors and treatment outcome through depression symptomatology, suggesting that the complex interplay between two psychologically related risk factors could be linked, rather than only directly, to the outcome of ART treatments. However, most importantly, when combined with previous data (moderating variables—H2) that indicated that the detrimental relationship between depression and treatment outcome was significantly buffered (moderated) by a positive attitude coping strategy, these findings suggest that it is possible to promote a counseling intervention that fosters disclosure and re-appraisal of one's own experiences to mitigate this detrimental relationship effectively.

Therefore, overall, the findings from this study provide an additional input to the relatively narrow branch of research that crosses the respective boundaries of the medical and psychological fields by identifying multiple predictors that can hinder (i.e., long duration of infertility/high number of treatment cycles, the social concern stressor, the avoiding coping strategy, State-Anxiety, and depression) or promote (i.e., the couple's relationship concern stressor, couple's dyadic adjustment, and the positive attitude coping strategy) ART treatment success. However, by adopting the transactional multidimensional perspective underpinning the I-STRESS model (8), the study also offers a further and more complex insight into the fact that infertile patients may be at higher or lower risk depending on the co-occurrence of multiple risk and/or protective factors. These should be comprehensively assessed and monitored throughout the infertility path in a joint effort of cooperation among healthcare professionals in the field of reproductive health (e.g., gynecologists/ obstetricians, reproductive endocrinologists, nurses, and psychologists).

Nonetheless, some limitations should be addressed. First, although the prospective design is considered valuable to establish a time sequence (in our case, starting from the beginning of infertility treatment) and indicate cause-and-effect relationships, inferences concerning the temporal associations between the predictors and ART treatment outcomes should be made with caution for several reasons. Indeed, whereas the inherent complexity of the interplay between psychological and medical health conditions hinders the possibility of establishing any causality, the study provided evidence of the statistically significant associations between psychological variables and treatment outcome. Moreover, further potential confounding factors that were not addressed in this study—both from the medical and psychological fields—may have influenced the outcome (e.g., adverse health behaviors) or may have occurred over the 4 years (e.g., worsening of medical conditions, further physical illness, changes in the type of pharmacological treatments, or other stressful live events such as loss of job and grief). In particular, this study did not cover the potential role of meaningful medical factors, such as the crucial parameters of reproductive health (e.g., BMI categories, hormone levels, and semen quality) or the type of ART treatment the patients had undergone. Future research should, therefore, be designed and developed by a multidisciplinary team to consider and explore the potential influence of a wider set of factors, from both the psychological and medical fields, that could potentially influence ART treatment outcomes. Furthermore, future research should be designed with more than two time-point assessments to provide more data and account for potential changes that could occur during the infertility path (e.g., new stressors such as changes in employment, medical interventions, and changes in medical parameters).

Moreover, despite the sample being large enough to explore the research hypotheses, a larger sample would strengthen the statistical power of the mediation/moderation analyses (particularly considering the limits of using dichotomized variables). Moreover, a larger and more heterogeneous sample would increase the generalizability of the findings. In particular, despite the evidence potentially being of international interest, the generalizability of the research results from this study is limited as the sample consisted of married white/Caucasian Italian women who were part of infertile heterosexual couples and diagnosed with primary infertility. This limits the generalizability of findings to other cultures, family structures, and patients with secondary infertility. Thus, future research should recruit a more heterogeneous sample and be designed with a cross-cultural design to strengthen the generalizability of the results and to make comparisons of findings beyond the national context.

### 4.1 Theoretical and clinical implications

Notwithstanding the abovementioned study limitations, the findings in this study provide an original contribution to the field of reproductive health, with specific theoretical and clinical implications. First, considering the strengths in the study design, the application of a statistically valid transactional and multidimensional predictive model sustains the reproducibility of this research design in other clinical settings. Furthermore, the use of the “live birth” indicator as the outcome, rather than a positive pregnancy test, allowed us to consider a treatment to be a success only when parenthood was achieved, thus emphasizing the need to go beyond the medical parameters alone when promoting reproductive health. In line with this, the recruitment of infertile women at the beginning of their treatment allowed us to explore the psychological impact of the infertility treatments on women whose emotional experiences were not yet affected by repeated treatment failures, and, accordingly, allowed us to define tailored evidence-based interventions to assist infertile patients throughout the infertility path.

In this direction, this study has several implications for clinical practice. Specifically, the adoption of a statistically valid framework could allow practitioners to routinely assess and monitor a wide set of psychological risk and protective factors that could potentially influence psychological adjustment among women undergoing ART treatments and potentially also be associated with treatment success. This framework also accounts for the complex interplay among psychological variables, thus reflecting the real-life experiences in which infertile patients can be exposed to multiple risk factors (e.g., the social concern stressor, greater use of the avoiding coping strategy, clinically relevant levels of anxiety and depression, an increased duration of infertility, and a high number of treatment cycles). However, at the same time, the patients could also possess (or, through tailored psychological support interventions, activate/strengthen) several individual (e.g., a positive reframing coping strategy) and dyadic resources to deal with the infertility experience and ART treatments.

However, beyond the implications for psychologists and psychotherapists working in the field of reproductive health, this study highlights the importance of adopting an integrated and multidisciplinary approach when planning and implementing treatment trajectories and interventions that target patients undergoing ART treatments. In this perspective, the evidence of the detrimental impact of repeated medical treatment cycles in a rapid succession underscores that all healthcare professionals (i.e., gynecologists, obstetricians, nurses, and psychologists), regardless of their discipline’s goals, should carefully balance and harmonize medical field evidence of how to maximize ART success rates with psychological evidence concerning the patients’ psychological needs, risks and available resources, with the specific goal of maximizing psychological adjustment to the infertility experience. Indeed, by plainly acknowledging the value of discussing, within multidisciplinary teams, moment-by-moment customized approaches for each patient, it could be possible to maximize and foster the goal of promoting the overall reproductive health of ART-treated patients, in terms of both psychological wellbeing and treatment success.

## Data Availability

The datasets will be provided by the corresponding author upon reasonable request. Requests to access these datasets should be directed to Maria Clelia Zurlo zurlo@unina.it.

## References

[1] Salari N Babajani F Hosseinian-Far A Hasheminezhad R Abdoli N Haydarisharaf P Global prevalence of major depressive disorder, generalized anxiety, stress, and depression among infertile women: a systematic review and meta-analysis. Arch Gynecol Obstet. (2024) 309(5):1833–4. 10.1007/s00404-024-07444-y 38459997

[2] Santamaría-Gutiez R Martínez-Corredor S González-Sala F Lacomba-Trejo L. Relevance of positive dyadic coping for couples undergoing assisted reproduction treatments: a systematic review. J Marital Fam Ther. (2025) 51(2):e70016. 10.1111/jmft.70016 40200679

[3] Shafaghi M Ahmadinezhad GS Karimi FZ Mazloum SR Golbar Yazdi HZ Afiat M. The effect of supportive counseling on self-esteem of infertile women after *in vitro* fertilization (IVF) failure: a randomized controlled trial study. BMC Psychol. (2024) 12(1):408. 10.1186/s40359-024-01914-3 39061102 PMC11282814

[4] Ombelet W Van Blerkom J Boshoff G Huyser C Lopes F Nargund G Now is the time to introduce new innovative assisted reproduction methods to implement accessible, affordable, and demonstrably successful advanced infertility services in resource-poor countries. Hum Reprod Open. (2025) 2025(1):hoaf001. 10.1093/hropen/hoaf001 39935763 PMC11810638

[5] World Health Organization (WHO). Infertility (2024). Available online at: https://www.who.int/news-room/fact-sheets/detail/infertility (Accessed July 14, 2024).

[6] Li G Jiang Z Kang X Ma L Han X Fang M. Trajectories and predictors of anxiety and depression amongst infertile women during their first IVF/ICSI treatment cycle. J Psychosom Res. (2021) 142:110357. 10.1016/j.jpsychores.2021.110357 33508704

[7] Molgora S Baldini MP Tamanza G Somigliana E Saita E. Individual and relational well-being at the start of an ART treatment: a focus on partners’ gender differences. Front Psychol. (2020) 11:2027. 10.3389/fpsyg.2020.02027 33117204 PMC7549400

[8] Zurlo MC Cattaneo Della Volta MF Vallone F. Infertility-related stress and psychological health outcomes in infertile couples undergoing medical treatments: testing a multi-dimensional model. J Clin Psychol Med Settings. (2020) 27(4):662–76. 10.1007/s10880-019-09653-z 31471847

[9] Zurlo MC Cattaneo Della Volta MF Vallone F. Re-examining the role of coping strategies in the associations between infertility-related stress dimensions and state-anxiety: implications for clinical interventions with infertile couples. Front Psychol. (2020) 11:614887. 10.3389/fpsyg.2020.614887 33414752 PMC7782436

[10] Zurlo MC Cattaneo Della Volta MF Vallone F. Paths towards parenthood after repeated treatment failures: a comparative study on predictors of psychological health outcomes in infertile couples persisting in treatments or opting for adoption. Front Psychol. (2023) 14:1147926. 10.3389/fpsyg.2023.1147926 37342643 PMC10277654

[11] Shingshetty L Cameron NJ Mclernon DJ Bhattacharya S. Predictors of success after *in vitro* fertilization. Fertil Steril. (2024) 121(5):742–51. 10.1016/j.fertnstert.2024.03.003 38492930

[12] Hazlina NH Norhayati MN Bahari IS Arif NA. Worldwide prevalence, risk factors and psychological impact of infertility among women: a systematic review and meta-analysis. BMJ Open. (2022) 12(3):e057132. 10.1136/bmjopen-2021-057132 35354629 PMC8968640

[13] Lopes HP. The integrated work between medicine and psychology with infertile couples at a public hospital in Rio de Janeiro/Brazil. JBRA Assist Reprod. (2022) 17(6):344–6. 35939552

[14] Boivin J Schmidt L. Infertility-related stress in men and women predicts treatment outcome 1 year later. Fertil Steril. (2005) 83(6):1745–52. 10.1016/j.fertnstert.2004.12.039 15950646

[15] Mirzaasgari H Momeni F Pourshahbaz A Keshavarzi F Hatami M. The relationship between coping strategies and infertility self-efficacy with pregnancy outcomes of women undergoing *in vitro* fertilization: a prospective cohort study. Int J Reprod Biomed. (2022) 20(7):539–48. 10.18502/ijrm.v20i7.11556 36187734 PMC9446440

[16] Imrie R Ghosh S Narvekar N Vigneswaran K Wang Y Savvas M. Socioeconomic status and fertility treatment outcomes in high-income countries: a review of the current literature. Hum Fertil (Camb). (2023) 26(1):27–37. 10.1080/14647273.2021.1957503 34315303

[17] Källén B Finnström O Nygren KG Olausson PO. *In vitro* fertilization in Sweden: maternal characteristics. Acta Obstet Gynecol Scand. (2005) 84(12):1185–91. 10.1111/j.0001-6349.2005.00858.x 16305706

[18] Liu X Shi J Mol BW Bai H. Impact of maternal education level on live birth rate after *in vitro* fertilization in China: a retrospective cohort study. J Assist Reprod Genet. (2021) 38(12):3077–82. 10.1007/s10815-021-02345-4 34694541 PMC8666395

[19] Mahalingaiah S Berry KF Hornstein MD Cramer DW Missmer SA. Does a woman’s Educational attainment influence *in vitro* fertilization outcomes? Fertil Steril. (2011) 95(8):2618–20. 10.1016/j.fertnstert.2011.05.015 21601850 PMC3925066

[20] Smith JF Eisenberg ML Glidden D Millstein SG Cedars M Walsh TJ Socioeconomic disparities in the use and success of fertility treatments: analysis of data from a prospective cohort in the United States. Fertil Steril. (2011) 96(1):95–101. 10.1016/j.fertnstert.2011.04.054 21616487 PMC3129357

[21] Wade JJ MacLachlan V Kovacs G. The success rate of IVF has significantly improved over the last decade. Aust N Z J Obstet Gynaecol. (2015) 55(5):473–6. 10.1111/ajo.12356 26174052

[22] Pottinger AM Nelson K McKenzie C. Stressful events and coping with infertility: factors determining pregnancy outcome among IVF couples in Jamaica. J Reprod Infant Psychol. (2016) 34(1):3–14. 10.1080/02646838.2015.1093613

[23] von Wolff M Schwartz AK Bitterlich N Stute P Fäh M. Only women’s age and the duration of infertility are the prognostic factors for the success rate of natural cycle IVF. Arch Gynecol Obstet. (2019) 299(3):883–9. 10.1007/s00404-018-5034-8 30607591

[24] Zarinara A Kamali K Akhondi MM. Estimation methods for infertility treatment success: comparison of four methods. J Fam Reprod Health. (2021) 15(3):179–85. 10.18502/jfrh.v15i3.7136 PMC8536827 34721609

[25] Glazener CM Ford WC Hull MG. The prognostic power of the post-coital test for natural conception depends on duration of infertility. Hum Reprod. (2000) 15(9):1953–7. 10.1093/humrep/15.9.1953 10966993

[26] Bodri D Kawachiya S De Brucker M Tournaye H Kondo M Kato R Cumulative success rates following mild IVF in unselected infertile patients: a 3-year, single-centre cohort study. Reprod Biomed Online. (2014) 28(5):572–81. 10.1016/j.rbmo.2014.01.002 24631167

[27] Malizia BA Hacker MR Penzias AS. Cumulative live-birth rates after *in vitro* fertilization. N Engl J Med. (2009) 360(3):236–43. 10.1056/NEJMoa0803072 19144939

[28] Karaca N Karabulut A Ozkan S Aktun H Orengul F Yilmaz R Effect of IVF failure on quality of life and emotional status in infertile couples. Eur J Obstet Gynecol Reprod Biol. (2016) 206:158–63. 10.1016/j.ejogrb.2016.09.017 27693938

[29] Gameiro S Finnigan A. Long-term adjustment to unmet parenthood goals: systematic review of long-term adjustment after failed fertility treatment. Hum Reprod Update. (2017) 23(3):322–37. 10.1093/humupd/dmx001 28164236

[30] Ni Y Tong C Huang L Zhou W Zhang A. The analysis of fertility quality of life and the influencing factors of patients with repeated implantation failure. Health Qual Life Outcomes. (2021) 19(1):32. 10.1186/s12955-021-01666-3 33494768 PMC7831164

[31] Zurlo MC Della Volta MFC Vallone F. Predictors of quality of life and psychological health in infertile couples: the moderating role of duration of infertility. Qual Life Res. (2018) 27(4):945–54. 10.1007/s11136-017-1781-4 29307056

[32] Cesta CE Johansson AL Hreinsson J Rodriguez-Wallberg KA Olofsson JI Holte J A prospective investigation of perceived stress, infertility-related stress, and cortisol levels in women undergoing *in vitro* fertilization: influence on embryo quality and clinical pregnancy rate. Acta Obstet Gynecol Scand. (2018) 97(3):258–68. 10.1111/aogs.13280 29250769

[33] Cooper BC Gerber JR McGettrick AL Johnson JV. Perceived infertility-related stress correlates with *in vitro* fertilization outcome. Fertil Steril. (2007) 88(3):714–7. 10.1016/j.fertnstert.2006.11.158 17316631

[34] McCarthy MP Chiu SH. Differences in women’s psychological well-being based on infertility treatment choice and outcome. J Midwifery Womens Health. (2011) 56(5):475–80. 10.1111/j.1542-2011.2011.00047.x 23181645

[35] Kitanovic B Tulic L Soldatovic I. Women coping strategies to infertility stress can impact IVF outcome EV1357. Eur Psychiatry. (2016) 33(S1):S624. 10.1016/j.eurpsy.2016.01.2342

[36] McLaughlin M Cassidy T. Psychosocial predictors of IVF success after one year: a follow-up study. J Reprod Infant Psychol. (2019) 37(3):311–21. 10.1080/02646838.2018.1560398 30585083

[37] Rapoport-Hubschman N Gidron Y Reicher-Atir R Sapir O Fisch B. “Letting go” coping is associated with successful IVF treatment outcome. Fertil Steril. (2009) 92(4):1384–8. 10.1016/j.fertnstert.2008.08.012 18930223

[38] Kakatsaki D Vaslamatzis G Chatziandreou M Anastasiadi K Dafni U Tzavara C Alexithymia is positively associated with the outcome of *in vitro* fertilization (IVF) treatment. Psychol Rep. (2009) 2(105):522–32. 10.2466/PR0.105.2.522-532 19928613

[39] Renzi A Solano L Di Trani M Ginobbi F Minutolo E Tambelli R. The effects of an expressive writing intervention on pregnancy rates, alexithymia and psychophysical health during an assisted reproductive treatment. Psychol Health. (2020) 35(6):718–33. 10.1080/08870446.2019.1667500 31549861

[40] Chen D Zhang JP Jiang L Liu H Shu L Zhang Q Factors that influence *in vitro* fertilization treatment outcomes of Chinese men: a cross-sectional study. Appl Nurs Res. (2016) 32:222–6. 10.1016/j.apnr.2016.07.003 27969032

[41] Renzi A Di Trani M Solano L Minutolo E Tambelli R. Success of assisted reproductive technology treatment and couple relationship: a pilot study on the role of romantic attachment. Health Psychol Open. (2020) 7:2055102920933073. 10.1177/2055102920933 32637146 PMC7323283

[42] Aimagambetova G Issanov A Terzic S Bapayeva G Ukybassova T Baikoshkarova S The effect of psychological distress on IVF outcomes: reality or speculations? PLoS One. (2020) 15(12):e0242024. 10.1371/journal.pone.0242024 33315878 PMC7735622

[43] Bapayeva G Aimagambetova G Issanov A Terzic S Ukybassova T Aldiyarova A The effect of stress, anxiety and depression on *in vitro* fertilization outcome in Kazakhstani public clinical setting: a cross-sectional study. J Clin Med. (2021) 10(5):937. 10.3390/jcm10050937 33804325 PMC7975982

[44] Haimovici F Anderson JL Bates GW Racowsky C Ginsburg ES Simovici D Stress, anxiety, and depression of both partners in infertile couples are associated with cytokine levels and adverse IVF outcome. Am J Reprod Immunol. (2018) 79(4):e12832. 10.1111/aji.12832 29528174

[45] Karlidere T Bozkurt A Ozmenler KN Ozsahin A Kucuk T Yetkin S. The influence of emotional distress on the outcome of *in vitro* fertilization (IVF) and/or intracytoplasmic sperm injection (ICSI) treatment among infertile Turkish women. Isr J Psychiatry Relat Sci. (2008) 45(1):55. 18587170

[46] Lintsen AM Verhaak CM Eijkemans MJ Smeenk JM Braat DD. Anxiety and depression have no influence on the cancellation and pregnancy rates of a first IVF or ICSI treatment. Hum Reprod. (2009) 24(5):1092–8. 10.1093/humrep/dep093 19176541

[47] Purewal S Chapman SC van den Akker OB. Depression and state anxiety scores during assisted reproductive treatment are associated with outcome: a meta-analysis. Reprod Biomed Online. (2018) 36(6):646–57. 10.1016/j.rbmo.2018.03.010 29622404

[48] Sohrabvand F Abedinia N Pirjani R Jafarabadi M. Effect of anxiety and depression on ART outcome. Iran J Reprod Med. (2008) 6:89–94.

[49] Zurlo MC Cattaneo Della Volta MF Vallone F. Factor structure and psychometric properties of the Fertility Problem Inventory–Short Form. Health Psychol Open. (2017) 4(2):2055102917738657. 10.1177/20551029177386 29379625 PMC5779934

[50] Sica C Magni C Ghisi M Altoè G Sighinolfi C Chiri LR Coping Orientation to Problems Experienced-Nuova Versione Italiana (COPE-NVI): uno strumento per la misura degli stili di coping. Psicoterapia Cogn Comportamentale. (2008) 14(1):27.

[51] Spanier GB. Measuring dyadic adjustment: new scales for assessing the quality of marriage and similar dyads. J Marriage Fam. (1976) 38(1):15–28. 10.2307/350547

[52] Gentili P Contreras L Cassaniti M D'arista F. La dyadic adjustment scale: una misura dell'adattamento di coppia. Minerva Psichiatr. (2002) 43:107–16.

[53] Pedrabissi L Santinello M. New Italian Version of the STAI-Form Y. Firenze, Italy: Giunti Organizzazioni Speciali (1989).

[54] Spielberger C. Anxiety: Current Trends in Research. London, UK: Academic Press (1972).

[55] Murray D Cox JL. Screening for depression during pregnancy with the Edinburgh Depression Scale (EDDS). J Reprod Infant Psychol. (1990) 8(2):99–107. 10.1080/02646839008403615

[56] Benvenuti P Ferrara M Niccolai C Valoriani V Cox JL. The Edinburgh postnatal depression scale: validation for an Italian sample. J Affect Disord. (1999) 53(2):137–41. 10.1016/S0165-0327(98)00102-5 10360408

[57] Hayes AF. Introduction to Mediation, Moderation, and Conditional Process Analysis: A Regression-based Approach. New York, NY: Guilford Publications (2017).

[58] Preacher KJ Hayes AF. “Contemporary approaches to assessing mediation in communication research”. In: Hayes AF Slater MD Snyder LB, editors. The Sage Sourcebook of Advanced Data Analysis Methods for Communication Research. Los Angeles, CA: Sage Publications, Inc. (2008). p. 13–54.

[59] Sobel ME. Asymptotic confidence intervals for indirect effects in structural equation models. Sociol Methodol. (1982) 1(13):290–312. 10.2307/270723

[60] Hayes AF Scharkow M. The relative trustworthiness of inferential tests of the indirect effect in statistical mediation analysis: does method really matter? Psychol Sci. (2013) 24(10):1918–27. 10.1177/0956797613480187 23955356

[61] Yassa M Arslan E Gulbahar DS. Effects of infertility treatment on anxiety and depression levels. Cukurova Med J. (2019) 44(2):410–5. 10.17826/cumj.456723

[62] Huang C Shi Q Xing J Yan Y Shen X Shan H The relationship between duration of infertility and clinical outcomes of intrauterine insemination for younger women: a retrospective clinical study. BMC Pregnancy Childbirth. (2024) 24(1):199. 10.1186/s12884-024-06398-y 38486148 PMC10938817

[63] Zanettoullis AT Mastorakos G Vakas P Vlahos N Valsamakis G. Effect of stress on each of the stages of the IVF procedure: a systematic review. Int J Mol Sci. (2024) 25(2):726. 10.3390/ijms25020726 38255800 PMC10815004

[64] Newton CR Hearn MT Yuzpe AA Houle M. Motives for parenthood and response to failed *in vitro* fertilization: implications for counseling. J Assist Reprod Genet. (1992) 9(1):24–31. 10.1007/BF01204110 1617245

[65] Rockliff HE Lightman SL Rhidian E Buchanan H Gordon U Vedhara K. A systematic review of psychosocial factors associated with emotional adjustment in *in vitro* fertilization patients. Hum Reprod Update. (2014) 20(4):594–613. 10.1093/humupd/dmu010 24676468

[66] Zurlo MC Cattaneo Della Volta MF Vallone F. The association between stressful life events and perceived quality of life among women attending infertility treatments: the moderating role of coping strategies and perceived couple’s dyadic adjustment. BMC Public Health. (2019) 19(1):1548. 10.1186/s12889-019-7925-4 31752817 PMC6873711

[67] Cassidy T McLaughlin M. Distress and coping with *in vitro* fertilisation (IVF): the role of self-compassion, parenthood motivation and attachment. J Psychol Clin Psychiatry. (2016) 6(4):00363. 10.15406/jpcpy.2016.06.00363

